# Molecular Electronics Meets Direct-Write Carbon Nanofabrication via Focused Electron-Beam-Induced Deposition (FEBID): A Platform for Junction Architecture Design

**DOI:** 10.1021/acsaelm.5c01566

**Published:** 2025-10-14

**Authors:** Aitor García-Serrano, Sara Sangtarash, Alejandro González-Orive, Hatef Sadeghi, Santiago Martín, Lucía Herrer, Richard J. Nichols, Paul J. Low, Colin J. Lambert, José María de Teresa, Soraya Sangiao, Pilar Cea

**Affiliations:** a Departamento de Física de la Materia Condensada, Escuela de Ingenieria y Arquitectura, 16765 Universidad de Zaragoza, Zaragoza 50009, Spain; b Instituto de Nanociencia y Materiales de Aragón (INMA), CSIC-Universidad de Zaragoza, Zaragoza 50009, Spain; c Department of Physics, 4396 Lancaster University, Lancaster LA1 4YB, U.K.; d School of Engineering, 2707 University of Warwick Coventry CV4 7AL, U.K.; e Instituto Universitario de Materiales y Nanotecnología, 16749 Universidad de La Laguna, San Cristobal de la Laguna 38200, Spain; f Laboratorio de Microscopias Avanzadas (LMA), 16765 Universidad de Zaragoza, Zaragoza 50018, Spain; g Department of Chemistry, 4591 University of Liverpool, Crown Street, Liverpool L69 7ZD, U.K.; h School of Molecular Sciences, University of Western Australia, Crawley, WA 6009, Australia; i Departamento de Física de la Materia Condensada, Facultad de Ciencias, 16765 Universidad de Zaragoza, Campus Plaza San Francisco, Zaragoza 50009, Spain

**Keywords:** electrografting, monolayer, FEBID, top contact, carbon electrodes, molecular junctions, 2D nanodevices, surface functionalization, charge transport

## Abstract

The electrical characteristics of a molecular junction are highly sensitive to the nature and uniformity of the molecule|electrode contacts. This gives rise to significant interest in the development of not only the active molecular structures that modulate charge transport and the anchor groups that contact them to the electrodes, but also methods for assembling uniform molecular monolayers on a substrate electrode and subsequent fabrication of a “top electrode” to achieve the reliable fabrication of viable molecular electronic devices. In this contribution, 4-(4-(4-(trimethylsilylethynyl)­phenylethynyl)­phenylethynyl)­aniline was converted to the corresponding diazonium salt and electrografted onto highly oriented pyrolytic graphite (HOPG), resulting in an organized monolayer covalently bonded to the HOPG “substrate” electrode. Subsequently, focused electron-beam-induced deposition was used to form an amorphous carbon top electrode (C-FEBID) onto the monolayer from a naphthalene precursor. By guiding the raster scanning of the electron beam, the position, shape, and thickness of the carbon electrode “written” onto the monolayer can be controlled with nanometer precision. In addition, as a proof-of-principle demonstration of the construction of the interconnects necessary for integration of molecular devices, platinum was deposited precisely on top of the C-FEBID electrodes, using focused-ion-beam-induced deposition of PtMe_3_Cp^Me^ (Cp^Me^ = η^5^-C_5_H_4_Me) (Pt-FIBID). The HOPG|molecule|C-FEBID|Pt-FIBID “large area” junctions produced in this manner exhibited excellent reproducibility and were free of short circuits for top-electrode dimensions ranging from 4 × 4 to 8 × 8 μm^2^. The electrical characteristics of these devices were measured and modeled by using quantum chemical approaches. These results illustrate alternative routes toward the fabrication of planar 2D devices based on molecular monolayers and carbon electrodes.

## Graphical abstract

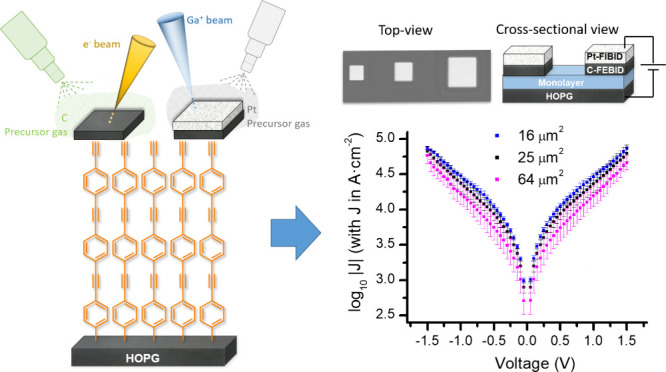

## 1 Introduction

The concepts of molecular electronics, in which one or more molecules located between two or three electrodes perform electrical functions, were first proposed in the 1950s and 1960s.^1^ Through remarkable advances in nanoscale engineering and scanning probe microscopy, these concepts have now been realized in molecular junctions formed from either single-molecules or “large area” arrays of molecules assembled into monolayer films.^2,3^ As understanding of electrical conductance mechanisms continues to grow, with phenomena such as room-temperature quantum interference now being recognized, researchers can not only rationalize but also deliberately tune the electrical behavior of molecular junctions for future applications.^4–6^ Consequently, molecular electronics may offer a viable alternative to our current CMOS (complementary metal-oxide-semiconductor) technology at extremely small scales or on flexible and biodegradable platforms^7^ and enable the development of novel computing paradigms (e.g., memory-based architectures) capable of performing complex computational tasks leading to the creation of molecular systems that mimic intelligent behavior,^8^ single-molecule sensing,^6^ and novel materials properties that are difficult to achieve in the solid state.^9^ Single-molecule junctions have been crucial in developing detailed understanding of molecular charge transport mechanisms.^10–13^

Nevertheless, in order for molecular electronic devices be a viable technology platform,^2^ there is a need to improve fabrication processes for the reliable, uniform construction of “large area” molecular junctions. Conceptually, the fabrication of a large-area junction involves the deposition of a uniform monolayer on a flat substrate electrode, for example, by self-assembly or Langmuir–Blodgett methods,^14–18^ followed by deposition of a “top-contact” electrode. The deposition of top contacts, in a manner that permits precise control of the location, size, and shape of the top contact and does not damage or short circuit the underlying monolayer, is a major focus of effort.^19^ Despite studies of large-area junctions and devices,^10,20,21^ a standard fabrication methodology that is compatible with contemporary device technologies remains elusive,^19^ largely due to the poor, ill-defined, and nonuniform molecule|electrode contacts formed at the monolayer-top-electrode interface.^22^ In addition, while gold serves as an important material for the exploration of molecular electronic phenomena as a substrate and/or top-contact electrode in both single-molecule and large-area junctions, the mobility of gold under large electric fields and sensitivity to local heating compromise its performance and stability, preventing its use in CMOS-style devices. There is an urgent need to develop “large area” molecular junction fabrication methodologies using techniques and materials that are compatible with commercial operation to realize the promises of molecular and electronic devices.

To these ends, carbon-based molecular electronic technologies, in which graphene, doped graphene, carbon nanotubes, graphite, amorphous carbon, or polymeric carbon-based materials are used as electrodes and active electronic elements,^7,23–25^ offer several key advantages, including simpler device processing and the fabrication of transparent, flexible, and stretchable devices, which are particularly attractive in the fields of optomolecular electronics and wearable devices.^26,27^ In addition, these technologies provide lower cost compared to gold, reduced environmental and e-waste impact, biocompatibility for medical applications, and tunable properties for diverse applications such as energy storage and biosensing.^25^ Among the pioneering contributions in the field of carbon-based molecular electronics, the work of McCreery deserves special mention,^28^ with the development of techniques for the grafting of a diazonium salt to modify a pyrolyzed photoresist film (PPF),^29^ electron-beam deposited carbon electrodes (e-carbon) on monolayers,^30^ and a subsequent “friendly process” for the deposition of gold on e-carbon,^30^ underpinned by theoretical modeling of carbon-based molecular electronic junctions, including their tunneling barrier profiles.^31^ McCreery’s group may also lay claim to the first commercialized molecular electronic device, in which a molecular rectifier composed of a molecular layer sandwiched between two carbon-based electrodes was used for audio processing applications.^32^ These achievements set the scene for the continued refinement of processes and strategies for assembly of a wider range of organic compounds between carbon-based electrodes and optimization of their electrical characteristics, with the ultimate goal of attaining electrical and material performance for real-word applications.

Motivated by the growing demand for scalable carbon-based molecular electronic platforms, we have sought to build upon our previous work on the fabrication of large-area carbon|molecule|carbon junctions using carbon-based focused-electron-beam-induced deposition (C-FEBID), in which an amorphous carbon top electrode of well-defined size, shape was directly written onto a Langmuir–Blodgett monolayer film supported on HOPG (highly oriented pyrolytic graphite),^33^ and other approaches for direct writing of conducting nanostructures.^34^ Here, we extend this strategy to demonstrate, for the first time, the integration of robust molecular monolayers electrografted onto HOPG substrate electrodes with the spatial precision and structural versatility offered by direct-write C-FEBID top electrodes. This direct-write approach has enabled the fabrication of covalently anchored carbon|molecule|carbon junctions in which the location and geometry of the top electrode are precisely defined over the substrate electrode bound monolayer. As a further step toward the construction of the nanoscale interconnects necessary for the integration of molecular components into device architectures, a platinum top-coat was subsequently layered precisely on the C-FEBID electrodes using focused-ion-beam-induced deposition of the metal from a PtMe_3_Cp^Me^ (Cp^Me^ = η^5^-C_5_H_4_Me) precursor (Pt-FIBID). In this contribution, we describe the characterization of each stage of the fabrication process, the physical and electrical properties of the resulting nascent devices, their potential for scalability, and the insights obtained through theoretical and computational modeling of these carbon-based molecular junctions.

## 2 Results and Discussion

The amine-functionalized oligo­(phenylene ethynylene) compound **1** (Figure 1) was converted to the corresponding diazonium salt and electrografted onto an HOPG surface following standard procedures (see details in the Supporting Information).^35–37^ The bulky trimethylsilyl (TMS) group incorporated into the molecular structure serves to impede dendritic three-dimensional growth during monolayer formation and ensure the formation of a well-ordered structure on the substrate, HOPG|**1** (Figure 2).^24^ Following successful grafting, as evidenced by the features displayed in the Raman spectra (Figure S2), the TMS group was cleaved by reaction with tetrabutylammonium fluoride solution in THF (verified by XPS analysis, Figure S2), yielding a monolayer bearing an exposed terminal ethynyl (C≡CH) functional group, denoted HOPG|**1′**. The modified electrode HOPG|**1′** was comprehensively characterized, which confirmed the formation of a well-ordered film, with a thickness of 2.1 ± 0.2 nm (Figure S3), in which the molecules are assembled into a tightly packed 2D arrangement with the molecular axis oriented nearly perpendicular to the HOPG surface and free of aggregates or other 3D defects on the surface and a very low roughness (as indicated by AFM imaging, Figure S4).^36^

**Figure 1:**
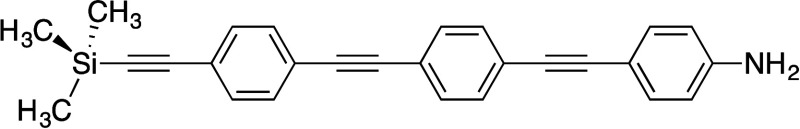
Chemical structure of 4-(4-(4-(trimethylsilylethynyl)­phenylethynyl)­phenylethynyl)­aniline (**1**).

**Figure 2:**
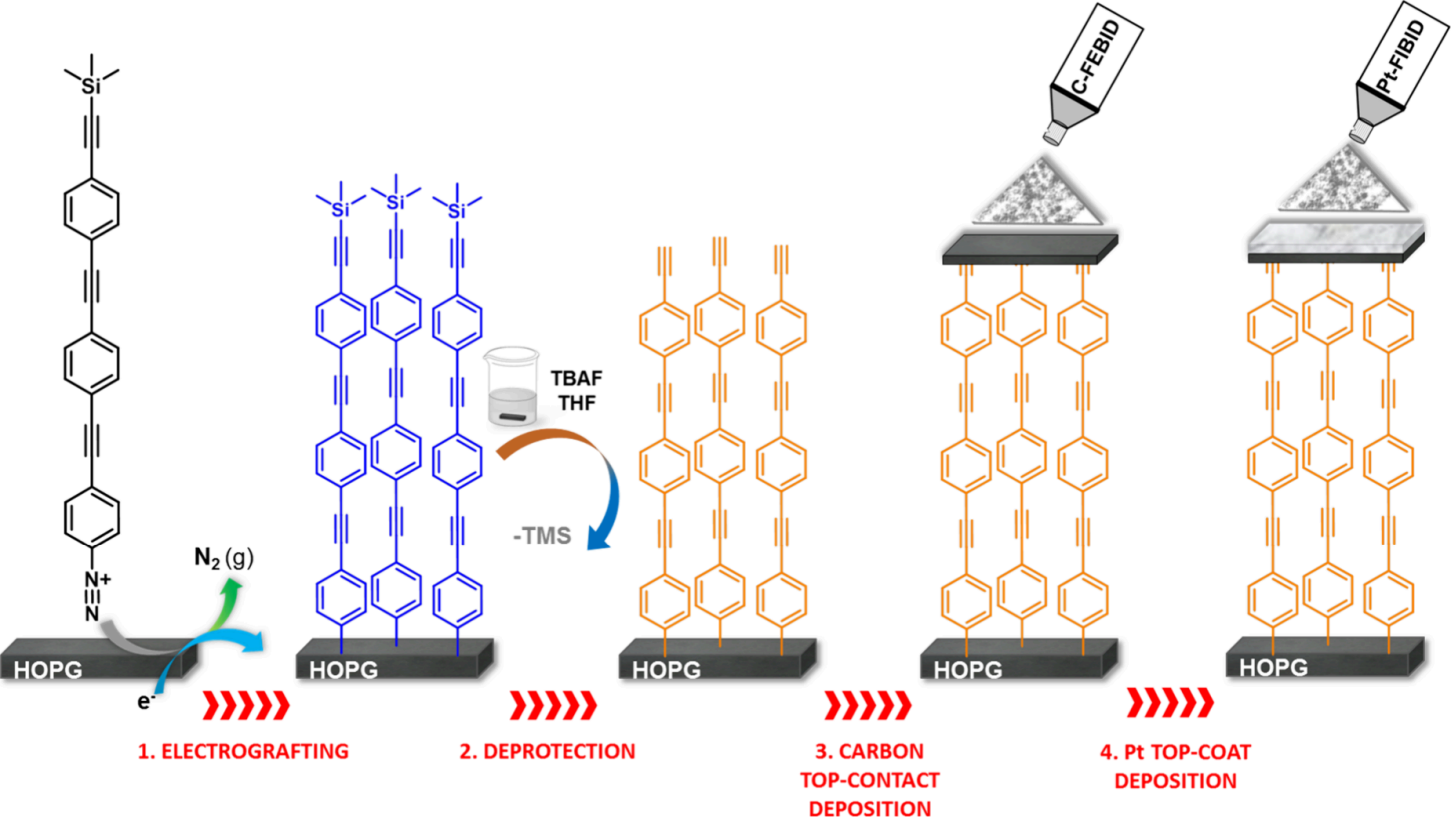
Schematic summary of the fabrication process described herein: (1) electrografting process of the diazonium salt formed *in situ* from compound **1** to give HOPG|**1**, (2) removal of the terminal TMS group by incubation of the monolayer in a TBAF/THF solution to give HOPG|**1′**, (3) deposition of the carbon-based top-contact electrode by C-FEBID, and (4) deposition of the platinum top-coat by Pt-FIBID.

Attention was subsequently turned to the deposition of a top-contact carbon electrode on top of HOPG|**1′** to create a large-area molecular junction. Focused electron-beam-induced deposition (FEBID) using gaseous naphthalene as a precursor has recently been shown to be an effective method for creating an amorphous carbon nanodeposit (C-FEBID) incorporating some graphitic crystallites.^33^ Naphthalene was selected for this work because aromatic hydrocarbons yield carbon-rich, conductive deposits with relatively high purity,^38^ in contrast to simpler aliphatic molecules such as ethylene, acetylene, or methane, which typically lead to deposits with higher hydrogen content and reduced electrical performance.^39,40^ While carbon top-contact electrodes have been deposited by other methods, such as electron-beam deposition of carbon from pure graphite using shadow masks,^30^ the C-FEBID has the following advantages: (i) shadow masks are not necessary to define the electrode pattern since, in this case, the pattern is directly written onto the monolayer in a shape and position controlled by the electron-beam scan with nm precision and without interpenetration or film damage (Figure 2);^33^ (ii) by exercising control over the precursor and e-beam, FEBID allows the shape, size, and thickness of the carbon deposit to be precisely determined; and (iii) the electrical properties of the resulting structures can be determined by contacting “*in situ*” to two electrical microprobes.

Using this FEBID method, a series of 50 nm thick carbon-based electrodes of various areas were “written” on top of a single HOPG|**1′** monolayer (Figure S5). Electron energy loss spectroscopy (EELS) served to demonstrate the absence of elemental impurities such as nitrogen and oxygen in these C-FEBID deposits (Figure S6). The ratio of the intensities of the D and G bands in the Raman spectra of these C-FEBID deposits (*I*(D)/*I*(G)) provides a quantitative measure of the amount of graphitic crystallites present in the carbon matrix: *I*(D)/*I*(G) = 0 corresponds to fully amorphous carbon, while *I*(D)/*I*(G) = 2.5 indicates fully nanocrystalline graphite (Figure S7).^41,42^ The observed value for the C-FEBID deposits, (*I*(D)/*I*(G) = 0.786, is consistent with an amorphous carbon structure that incorporates some graphitic crystallites.^42–44^

The slightly *S*-shaped *J–V* curve observed in the control junction experiment (Figure S8) reflects the intrinsically modest conductivity of the as-deposited C-FEBID top electrode, which typically consists of amorphous hydrogenated carbon with a carbon content of 70–80%.^45^ The relatively low in-plane conductivity of amorphous carbon and resulting ohmic potential losses may be significant when the nascent “large area” HOPG|**1′**|C-FEBID junction is connected to the external circuit for electrical measurements. To improve both the accuracy and reproducibility of the junction measurements and the electrical contact efficiency with the carbon structure, and indirectly with the underlying molecular monolayer, C-FEBID electrodes were overcoated with a 130 nm thick Pt-based layer using a similar focused *ion beam*-induced deposition method (Pt-FIBID) (Figure S5). In a manner closely related to FEBID, in FIBID, the gaseous precursor (here trimethyl­(methylcyclopentadienyl)­platinum, PtMe_3_CpMe) is delivered onto the surface and decomposed by a focused gallium ion beam on the surface (Figure 2). Focused-ion-beam scanning dissociates the precursor gas molecules and creates a platinum-based nanodeposit. The resistivity of Pt deposited by FIBID is typically 4 orders of magnitude lower (i.e., the conductivity is 4 orders of magnitude higher) than that of similar platinum structures deposited by FEBID,^46^ making FIBID the preferred method for this application. These differences arise from the distinct fragmentation pathways of PtMe_3_CpMe under electron and ion irradiation: FEBID typically yields carbon-rich deposits with high resolution, whereas FIBID provides higher Pt content and conductivity at the expense of slightly lower resolution.^46^ Moreover, recent advances in theoretical and computational modeling have provided new insights into these process. In particular, irradiation-driven molecular dynamics approaches^47^ allow atomistic simulation of precursor fragmentation under different irradiation conditions, thereby helping to rationalize the experimentally observed differences in morphology between FEBID and FIBID. The overlay control over two FEBID or FIBID nanofabrication steps inside the Dual Beam chamber is excellent and, usually, only limited by electronic drifts during the deposition process: e.g., thermal drifts, environmental noise, charge accumulation on the substrate or the previous deposit, etc. This precise spatial registry over two deposition steps is a prerequisite for future device fabrication and CMOS compatibility. As a result of this two-step fabrication process, each single “large area” junction has a dual top layer C-FEBID/Pt-FIBID contact of controlled composition and well-defined area specified by the raster path of the electron and ion beams.

Several HOPG|**1′**|C-FEBID|Pt-FIBID devices with top-contact areas ranging from 4 × 4 to 8 × 8 μm^2^ were fabricated on the same HOPG|**1′** substrate (Figure 3a). The electrical properties of the resulting “large area” junctions were determined by contacting the top C-FEBID|Pt-FIBID electrode and the bottom HOPG electrode using microprobes and measuring the current in response to applied bias. Twenty-three devices fabricated on different regions of two separately formed HOPG|**1′** systems were electrically characterized in this manner, none of which exhibited any electrical behavior that could be attributed to the formation of short circuits (Figure 3b). In each case, the measured *J*–*V* curves exhibit a similar shape, although there is a tendency for larger devices to exhibit a lower conductance, Figure 3c, which is more clearly visible in plots of the mean log_10_|*J*| values vs *V* (Figure 3d). This effect can be tentatively attributed to a decrease in the effective number of molecules that make electrical contact with the electrode as the area of the device increases.^48,49^ Accordingly, although the reproducibility is satisfactory for the smaller junctions, it becomes more challenging in the largest devices (64 μm^2^), where the probability of local defects within the molecular monolayer increases, leading to a broader dispersion of the current density values. This interpretation is supported by the observation that devices with 4 × 4 μm^2^ areas exhibit substantially smaller error bars compared to larger ones, suggesting that their effective contact area is closer to the nominal value, whereas deviations become increasingly significant as the nominal area grows, thereby increasing variability among devices. Control experiments to rule out any Schottky behavior or other electrical artifacts from the electrodes and material structures in the absence of the molecular monolayer were also performed (Figure S8). Importantly, Simmons fittings^50^ of the *J*–*V* curves (Equation S1) yield barrier thicknesses of 2.08–2.15 nm, in excellent agreement with the AFM measurements (2.1 ± 0.2 nm; Figure S3) for the grafted monolayer prior to the top-contact electrode deposition (see Figure S9 and the associated discussion in the Supporting Information).

**Figure 3:**
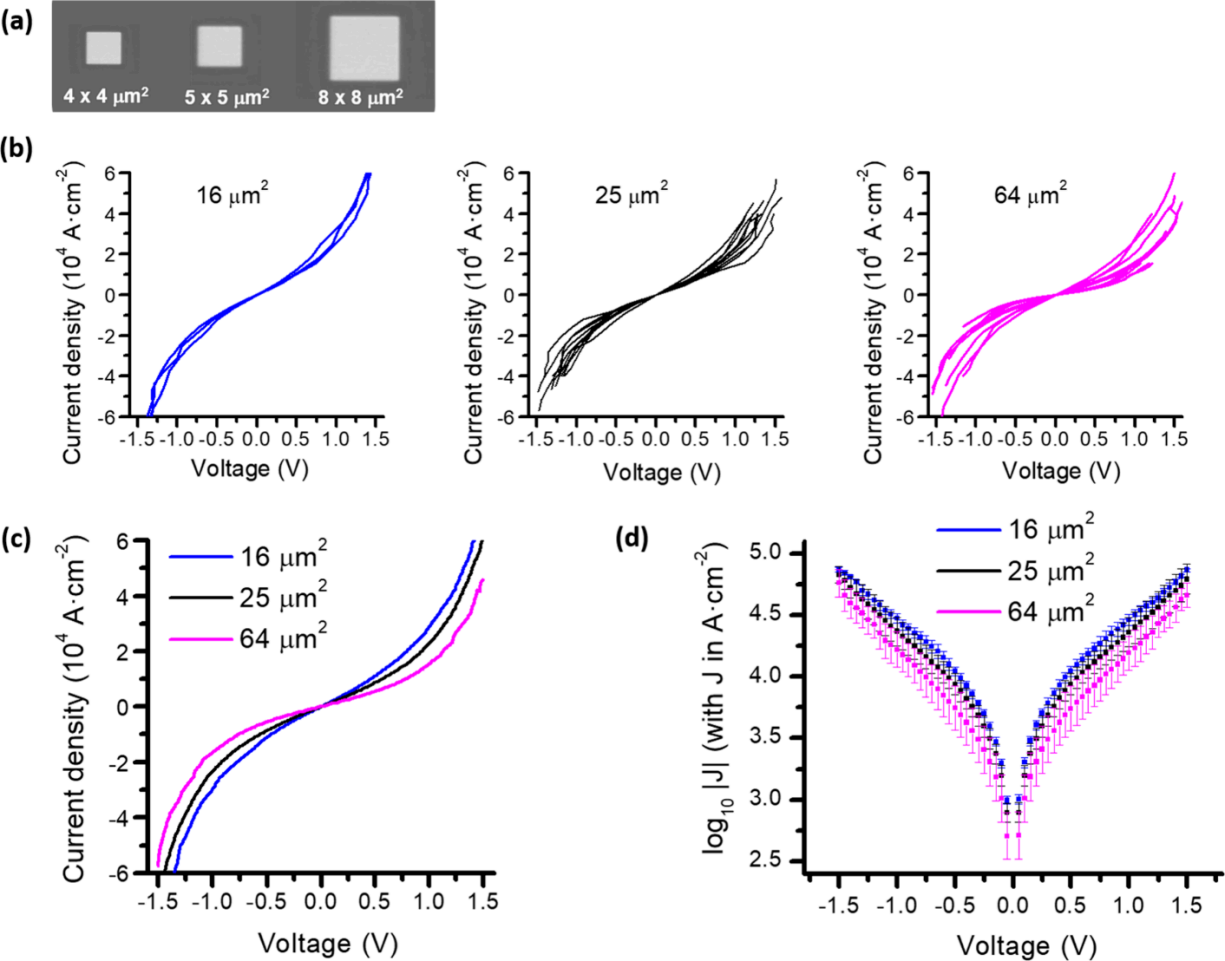
(a) Scanning electron micrograph of representative HOPG|**1′**|C-FEBID|Pt-FIBID devices (50 nm thick C-FEBID|130 nm thick Pt-FIBID layers). (b) *J–V* characteristics of 23 different HOPG|**1′**|C-FEBID|Pt-FIBID molecular electronic devices with nominal contact areas of 16 μm^2^ (blue curves), 25 μm^2^ (black curves), and 64 μm^2^ (magenta curves). (c) Comparison of average *J*–*V* characteristics for devices with different junction areas (16, 25, and 64 μm^2^). (d) Mean log_10_|*J*| values (squares) and standard deviation (error bars) for each device size using the same color coding as in panels (b) and (c).

To model electron transport through molecules beneath the C-FEBID and Pt-FIBID layers, quantum transport calculations using a material-specific Hamiltonian obtained from the SIESTA implementation of density functional theory (DFT) were carried out on a range of possible single-molecule junction structures and contacts (Figure 4 and Figure S10).^51^ For a given structure, these calculations yield the transmission coefficient *T*(*E*) describing electrons of energy *E* passing from one electrode to the other via a molecule, from which the electrical conductance is obtained using the Landauer formula. As discussed above, the C-FEBID deposits are hydrogenated amorphous carbon,^33,41,52^ and therefore, the C-FEBID electrode in contact with the molecular species was modeled as a graphene-like layer, with several types of defects close to the connection point to the molecule. Although the predicted transport properties are sensitive to the precise binding configuration employed in the model, as might be reasonably expected, the average of the transmission coefficients of junctions J1–J4 near the middle of the HOMO–LUMO gap is about 3 × 10^–6^ G_0_ (Figure 4). Given the approximations involved, this is in remarkably good agreement with estimates of the single-molecule conductance values, ca. 1 × 10^–6^ G_0_, drawn from the experimental large-area devices, with area 5 × 5 μm^2^ and assuming a molecular density over the surface of 3.6 × 10^–10^ mol·cm^–2^ (or ca. 2.2 × 10^14^ molecules·cm^–2^). It also compares with experimental single-molecule conductance data for OPE derivatives with three aromatic rings and different anchoring groups sandwiched between a metal substrate electrode and a metallic STM tip (which generally fall the range 10^–4^ to 10^–5^ G_0_).^11,15^

**Figure 4:**
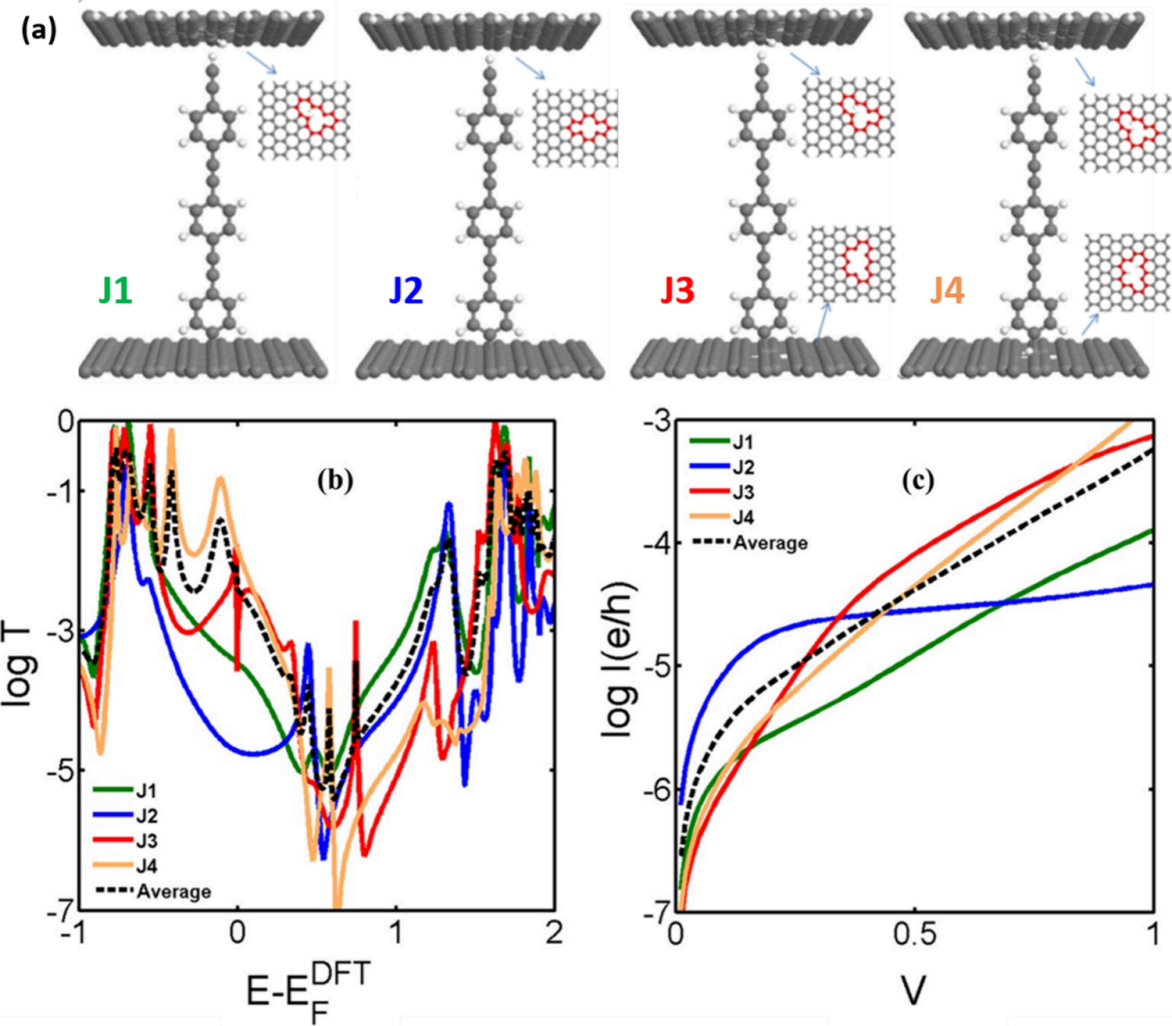
(a) Figure representing the relaxed structures of the model junctions: in J1 and J2, the molecular species **1′** is covalently bound to a pristine graphene-like model of the HOPG bottom electrode while the top contact is modeled as a 2D carbon layer containing saturated and unsaturated carbon defect sites, respectively, and physisorbed to the terminal alkyne (C≡CH); in J3 and J4, unsaturated and saturated defect sites are introduced to the HOPG model bottom electrode, respectively, while the top contact is a 2D carbon layer containing saturated carbon defect sites physisorbed to the terminal alkyne (C≡CH). Plots of the: (b) transmission coefficients, and (c) calculated current–voltage plots from the model junctions J1–J4.

Previous work has consistently shown a significantly lower conductance per molecule in large-area devices compared to single-molecule junctions, up to a factor of 10^8^ in large-area molecular junctions consisting of 10^3^ to 10^12^ molecules/active device area (our devices are in the order of 10^7^ to 10^8^ molecules for the μm^2^ device areas prepared here).^48,53–55^ This discrepancy between molecular conductance determined directly from single-molecule measurements and estimated from large-area junctions has been mainly attributed to poor electrical contact of the monolayer with the electrodes in the large-area devices.^54,56–58^ In some cases, correction factors accounting for differences between the effective electrical area and the geometric area of the electrode have been introduced to reconcile experimental observations.^59^ However, other examples of well-contacted monolayers exhibiting conductance values, which can be compared to single-molecule junctions when accounting for different electrode materials, include the gold|biphenyl-4,4′-dithiol SAM|graphene junctions prepared by Li et al.,^48^ where the experimental conductance (2.4 × 10^–^^3^ nS) compares with the single-molecule value measured between two gold electrodes (1.82 nS). Agreement here can be reconciled by considering that DFT modeling predicts the conductance between gold and graphene electrodes to be 10^2^ to 10^3^ times lower than that between two gold electrodes, which then brings these two values into reasonable alignment.^48^ Examples of efficient devices with effective molecule|electrode contacts that most closely map to single-molecule data include large-area gold|*n*-alkanethiol monolayer|graphene|gold devices, which exhibited resistances per molecule consistent with those of single-molecule metal|molecule|metal junctions;^60^ gold|*n*-alkanethiol monolayer|PEDOT:PSS|gold devices, in which the current per molecule was comparable to that of benchmark nanopore diodes;^61^ and the work by Karuppannan et al., who reported Au|*n*-alkanethiolate SAM|carbon-paint|Au devices exhibiting high current densities comparable to single-molecule junctions.^20^ The conductance per molecule estimated from the large-area HOPG|electrografted OPE|C-FEBID|Pt-FIBID devices prepared here (ca. 10^–6^ G_0_) is only 1–2 orders of magnitude lower than the single-molecule conductance values typically reported for OPE derivatives with similar backbones and various anchoring groups in metal|molecule|metal configurations (10^–^^4^ to 10^–^^5^ G_0_).^11,15^ This relatively small difference between the molecular conductance determined from our large-area and single-molecule device values highlights the quality of the electrical contact and effectiveness of our interface design.

The performance and high yield of the HOPG|electrografted OPE|C-FEBID|Pt-FIBID devices represent a promising proof-of-concept for the fabrication of efficient large-area molecular junctions and are attributed here to the combined contributions and synergies of several factors: (i) an electrografting process that ensures a robust anchoring and chemisorption of the material on the bottom electrode, resulting in stable and covalent bonding that contributes to a more defined and reproducible interface; (ii) the presence of carbon-based electrodes that minimize atomic diffusion processes into the molecular monolayer, typically associated with conventional metal electrodes, especially under electrical bias or thermal stress upon device operation; and (iii) a top-contact approach that results in a high yield without damaging the underlying monolayer or causing short circuits, thereby preserving its structural and electronic integrity. Altogether, these phenomena result in improved interfacial quality and enhanced charge transport across the large-area junction, with conductance per molecule much larger than most devices found in the literature. This approach offers remarkable nanofabrication advantages, including not only the high yield and high current densities but also the precise deposition of the top-contact electrode directly onto targeted locations, with the desired shape and size through direct-write techniques, eliminating the need for masks that would otherwise complicate the process and reduce accuracy. In addition, interconnects could also be fabricated within the same FEBID/FIBID chamber, further streamlining the workflow.

## 3 Conclusions

The electrografting of a diazonium-functionalized oligo­(phenylene ethynylene) (OPE) derivative onto HOPG has enabled the formation of a densely packed, covalently anchored monolayer with high structural integrity. Building upon this robust molecular platform, we have developed a route for the fabrication of large-area molecular junctions via the direct deposition of carbon-based top electrodes using focused electron-beam-induced deposition (C-FEBID). Importantly, this strategy allows the construction of electrodes with desired sizes, shapes, and thicknesses, providing exquisite control over the final device architecture. The use of a complementary focused-ion-beam strategy (Pt-FIBID) to overlay a platinum layer precisely on the C-FEBID pad further enhances the electrical performance of the junctions without compromising the molecular monolayer beneath. Together, these nanofabrication strategies provide a route to reproducible electrical contacts with nanometric spatial precision while minimizing perturbation of the active molecular layer.

The ability to deposit both carbon and metallic electrode materials with nanoscale precision through FEBID and FIBID technologies and complete registry onto molecular monolayers offers a powerful entry point for advancing molecular electronics. This approach enables not only the formation of stable molecular contacts but also the integration of functional interconnects, opening new directions for the bottom-up construction of potentially scalable, high-performance molecular electronic devices. Looking ahead, these findings have significant implications for the development of the next-generation of molecular electronic devices. The potential to create devices that bridge molecular-scale architectures with macroscopic functionality is enormous, and this work represents a step toward realizing this vision.

## 4 Methodology

### 4.1 Materials and Reagents

Compound **1** was synthesized by literature methods.^62^ Anhydrous acetonitrile was purchased from Sigma-Aldrich (99.9%). Tetraethylammonium tetrafluoroborate (NEt_4_BF_4_) (≥99.0%, Sigma-Aldrich) was dried at 80 °C in vacuum for 24 h before use. The compounds *tert*-butylnitrite (90%, Sigma-Aldrich), ferrocyanide (≥99%, Scharlau), and dopamine (3,4-dihydroxiphenylethylamine) (≥99.9%, Sigma-Aldrich) were purchased and used as received. The redox probes together with the corresponding electrolytes, either 0.1 M NEt_4_BF_4_ or 0.1 M KCl (99% from Fluka), 0.1 M KClO_4_ and 0.1 M H_2_SO_4_, were dissolved in acetonitrile or Millipore Milli-Q water (resistivity 18.2 MΩ·cm), respectively. SPI-2 quality-grade HOPG substrates were purchased from SPI supplies.

### 4.2 *In Situ* Generation of the Diazonium Salt^35,63^

Electrografted films of **1′** were fabricated in an electrochemical cell containing an acetonitrile solution of the reducing agent *tert*-butyl nitrite (7.5 mM), NEt_4_BF_4_ (supporting electrolyte and reagent, 0.1 M), and compound **1** (2.5 mM). The reaction was allowed to progress for 30 min under stirring and with a nitrogen flux. More details can be found in the Supporting Information.

### 4.3 Electrochemical Methods

All electrochemical measurements were undertaken by using an Autolab PGSTAT302N potentiostat (Metrohm-Autolab, BV, The Netherlands). The counter electrode (CE) was a Pt sheet. The reference electrode employed in the electrografting experiments was a nonaqueous Ag/Ag^+^ (0.01 M AgNO_3_ in acetonitrile) electrode purchased from BAS, calibrated versus the redox potential of Fc/Fc^+^ redox probe (*E*_1/2_(Fc/Fc^+^) = 0.090 V versus Ag/Ag^+^). The working electrode was an HOPG substrate freshly cleaved using scotch tape prior to its use. The electrografted films were obtained by repetitive (up to 3 cycles) cycling between 0.4 and −0.8 V at 50 mV·s^–1^. After every single scan, the grafted HOPG electrode was thoroughly rinsed with acetonitrile and sonicated in an acetonitrile, ethanol, and acetone bath for 5 min in each solvent to remove the physisorbed material. After that, samples were dried under a nitrogen flux (and returned for the second cycle). All electrode potentials quoted in the text are referred to the Ag/Ag^+^ (0.01 M AgNO_3_ in acetonitrile) reference scale when voltammograms were conducted in acetonitrile and to Ag/AgCl (3 M KCl) when the electrochemical response was measured in aqueous solution.

### 4.4 Deprotection of Silyl Groups

To remove trimethylsilyl (TMS) protecting groups, monolayers of **1′** were dipped into a solution containing 50 mM tetrabutylammonium fluoride (TBAF) in THF for 90 min. The as-treated samples were thoroughly rinsed in THF, EtOH, and acetone, and finally, they were dried under a nitrogen flux.

### 4.5 Top-Contact Electrode

The C-FEBID/Pt-FIBID electrode was deposited by means of an FEI dual beam instrument, which combines a 30 kV scanning electron microscope (SEM) and a 30 kV focused ion beam. These sit inside a process chamber at 52° giving a coincidence point and defining the working area. The carbon-based layer was grown by using a focused-electron-beam energy of 5 kV and a beam current of 26 nA, and the Pt-based layer was grown by using a focused-ion-beam energy of 30 kV and a beam current of 0.2 nA.

### 4.6 Characterization

AFM imaging was achieved in both Tapping and Peak-Force modes by using a Multimode 8 microscope in conjunction with a Nanoscope V control unit, both from Bruker. The microscope was operated in ambient air conditions using a scan rate of 0.5–1.2 Hz. To this end, RFESPA-75 (75–100 kHz, and 1.5–6 N·m^–1^, nominal radius of 8 nm) and ScanAsyst-Air-HR (130–160 kHz, and 0.4–0.6 N·m^–1^, nominal radius of 2 nm) tips, purchased from Bruker, were used. Nanoscope off-line v. 1.40 and Gwyddion v. 2.41 package softwares were used for the determination of the RMS roughness and depth statistical analysis. X-ray photoelectron spectroscopy (XPS) spectra were obtained with a Kratos AXIS ultra DLD spectrometer fitted with a monochromatic Al Kα X-ray source (1486.6 eV) and using a pass energy of 20 eV. The XPS binding energies here presented were all referenced with respect to the C 1s peak at 284.6 eV, which provides the required energy calibration. Raman spectra were recorded using a Confocal Raman Imaging Microscope with an excitation wavelength of 633 nm from Witec (model Alpha300M+). An EELS experiment was performed in an FEI Titan 60-300 transmission electron microscope operated at 300 kV and fitted with a high brightness electron gun (X-FEG) and a C_S_ probe corrector (CETCOR), which produces an electron probe below 1 Å in STEM, and a Gatan Imaging Filter (GIF) Tridiem 866 ERS. EELS spectra acquisition was performed with an energy dispersion of 0.2 eV·pixel^–1^, with a resolution of 0.9 eV (fwhm of the zero-loss peak), and a GIF aperture of 6 mm to provide a collection angle of 55 mrad. To minimize possible beam damage, the spectra were acquired with a 40 s exposure while scanning a sample area of 10 × 10 nm^2^.

### 4.7 Electrical Measurements

The electrical properties of the devices fabricated in this study were recorded by contacting them *in situ* with two electrical microprobes from Kleindiek. These microprobes were connected via a feed-through to a Keithley system featuring a combination of 6220 DC current source and a 2182 nanovoltmeter, both situated on the outside of the microscope chamber.

### 4.8 Computational Studies

The optimized geometry and ground-state Hamiltonian and overlap matrix elements of each structure studied in this paper were self-consistently obtained using the SIESTA^51^ implementation of density functional theory (DFT). SIESTA employs norm-conserving pseudopotentials to account for the core electrons and linear combinations of atomic orbitals (LCAO) to construct the valence states. The real-space grid is defined with an equivalent energy cutoff of 250 Ry. The geometry optimization for each structure is performed to the forces smaller than 20 meV Å^–1^. For transport calculations, the generalized gradient approximation (GGA) of the exchange and correlation functional was used with the Perdew–Burke–Ernzerhof (PBE) parametrization and a double-ζ polarized (DZP) basis set. The mean-field Hamiltonian obtained from the converged SIESTA DFT calculations was combined with our implementation of the nonequilibrium Green’s function method, the Gollum,^64^ to calculate the phase-coherent, elastic scattering properties of the each system consisted of left (source) and right (drain) carbon-based leads connected to the scattering region formed from monomer wires with different structures. The transmission coefficient^65^ *T*(*E*) for electrons of energy *E* (passing from the source to the drain) is calculated via the relation *T*(*E*) = trace­(Γ_*R*_(*E*)*G* ^*R*^(*E*)­Γ_*L*_(*E*)*G* ^*R*†^(*E*)). In this expression, $\Gamma_{L,R}(E)=i\left(\sum_{L,R}(E)-{\sum_{L,R}}^{†}(E)\right)$ describes the level broadening due to the coupling between left (*L*) and right (*R*) electrodes and the central scattering region, ∑_*L*,*R*_(*E*) contains the retarded self-energies associated with this coupling, and *G* ^*R*^ = (*ES* – *H* – ∑*_L_* – ∑*_R_*)^−1^ is the retarded Green’s function, where *H* is the Hamiltonian and *S* is the overlap matrix. Using obtained transmission coefficient *T*(*E*), the conductance could be calculated using the Landauer formula *G* = *G*_0_ ∫ *dE T*(*E*)­(−∂*f*(*E*, *T*)/∂*E*), where *G*_0_ = 2*e* ^2^/*h* is the conductance quantum, *f*(*E*) = (1 + exp ((*E* – *E*_*F*_)/*k*_B_ *T*))^−1^ is the Fermi–Dirac distribution function, *T* is the temperature, and *k*_B_ = 8.6 × 10^–5^ eV K^–1^ is Boltzmann’s constant.

## Supplementary Material



## Notes

Electrografting procedure; XPS and Raman spectroscopy data; film thickness and RMS roughness measurements; AFM, EELS, and Raman characterization of C-FEBID top contact; control experiments for *J*–*V* curves; Simmons model fittings; and details of theoretical calculations (PDF)
