# Supplementary material for: Molecular Electronics Meets Direct-Write Carbon Nanofabrication via Focused Electron-Beam-Induced Deposition (FEBID): A Platform for Junction Architecture Design
Source: ACS Appl Electron Mater. 2025 Oct 14;7(20):9470–9. doi: 10.1021/acsaelm.5c01566 (PMC12573786; doi:10.1021/acsaelm.5c01566)
Supplement: Supplementary file 1 [file el5c01566_si_001.pdf]

Supporting Information for

Molecular Electronics Meets Direct-Write Carbon Nanofabrication via Focused Electron Beam Induced Deposition (FEBID): A Platform for Junction Architectures Design

Aitor García-Serrano,[a,b‡] Sara Sangtarash,[c,d‡*] Alejandro González-Orive,[e] Hatef Sadeghi,[c,d] Santiago Martín,[a,b,f] Lucía Herrer,[b] Richard J. Nichols,[g] Paul J. Low,[h] Colin J. Lambert,[c*] José María de Teresa,[b] Soraya Sangiao,[b,f,i*] Pilar Cea,[a,b,f*]

[a] Departamento de Química Física, Facultad de Ciencias, Universidad de Zaragoza, 50009, Zaragoza, Spain.

[b] Instituto de Nanociencia y Materiales de Aragón (INMA), CSIC-Universidad de Zaragoza, 50009 Zaragoza, Spain.

[c] Department of Physics, Lancaster University, Lancaster LA1 4YB, UK.

[d] School of Engineering, University of Warwick, Coventry, CV4 7AL, UK

[e] Instituto Universitario de Materiales y Nanotecnología, Universidad de La Laguna, San Cristobal de la Laguna, 38200, Spain.

[f] Laboratorio de Microscopias Avanzadas (LMA), Universidad de Zaragoza, 50018, Zaragoza, Spain.

[g] Department of Chemistry, University of Liverpool, Crown Street, Liverpool, L69 7ZD, United Kingdom.

[h] School of Molecular Sciences, University of Western Australia, Crawley, 6009, WA, Australia.

[i] Departamento de Física de la Materia Condensada. Facultad de Ciencias. Universidad de Zaragoza. Campus Plaza San Francisco, 50009 Zaragoza, Spain.

‡These two authors contributed equally to this work

*[illegible]

Table of Contents

1. Electrografting process 3
2. XPS 3
3. Raman spectroscopy 5
4. Film thickness 5
5. RMS Roughness 6
6. AFM Characterization of C-FEBID Top Contact and Pt-FIBID Top Coat 8
7. EELS Characterization of C-FEBID Top Contact 9
8. RAMAN Characterization of C-FEBID Top Contact 10
9. Control Experiments in the *J-V* curves 10
10. Simmons Fittings 11
11. Theoretical Calculations 12
12. References 15

1. Electrografting process

The amine-functionalised oligo(phenylene ethynylene) compound **1** (Figure 1 in the manuscript), 2.5 mM solution in acetonitrile, was reacted with nitrosyl fluoride (NOF) in acetonitrile solution, 7.5 mM, to form the diazonium derivative *in situ,* before being electrografted onto a HOPG electrode by ramping the potential from +0.4 to –0.8 V (vs Ag/Ag^{+}; 0.01 M $AgNO_3$ in acetonitrile) at 50 $mV{\cdot}s^{-1}$ (Scheme 1 in the manuscript). The bulky trimethylsilyl (TMS) group serves to prevent the monolayer from dendritic three-dimensional growth, resulting in a well-formed monolayer of uniform thickness covalently bonded to the HOPG surface.[1]

After electrografting, the TMS group was removed by treatment with fluoride (as a solution of the tetrabutylammonium, TBA^{+}, salt) to give the modified monolayer bearing an exposed ethynyl (C≡CH) functional group on the top surface of the carbon-grafted monolayer, denoted HOPG|**1'** (Figure 2 in the manuscript), allowing for subsequent integration into molecular junction architectures.

2. XPS

X-ray Photoelectron Spectroscopy (XPS) was used to verify the electrografting process of compound **1** by the disappearance of the $–NH_2$ terminal group as well as the efficient deprotection of the TMS group by the disappearance of the Si signal after incubation of HOPG/monolayer of **1´** in TBAF, Figure S1.

The characteristic N1*s* peak (observed from samples of **1** at 399.2 eV) was absent from the spectrum recorded from HOPG|**1'**, indicating the absence of unreacted amine, protonated ammonium, or diazonium ions derived from **1** which could have been incorporated into the monolayer or physisorbed onto the graphite surface.[2] Similarly, the absence of Si2*p* signals in the HOPG|**1´** spectrum indicates the complete removal of the TMS protecting group.

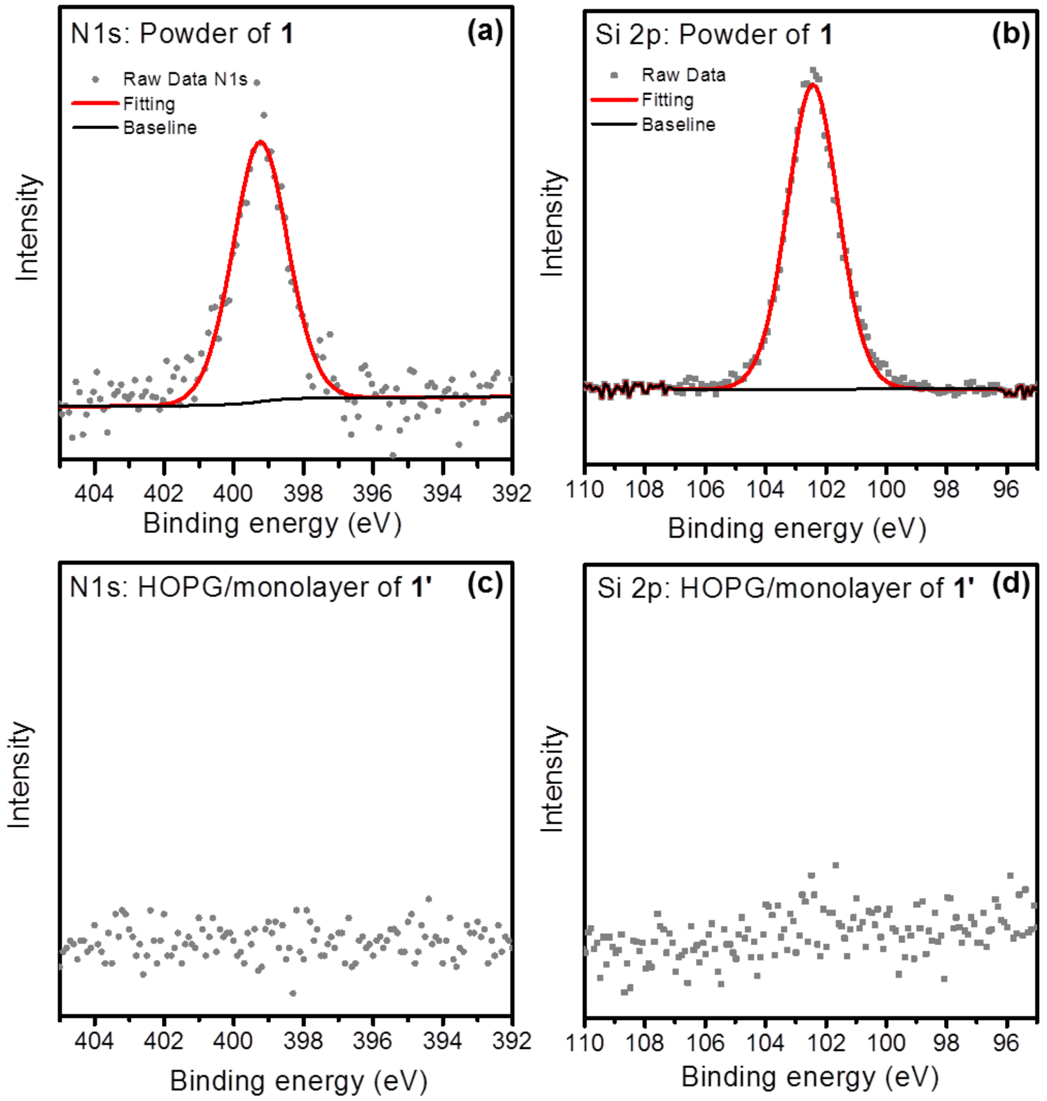

Figure S1. XPS spectra of the **(a)** N1s region for the powder of **1**; **(b)** Si2p region for the powder of **1**; **(c)** N1s region for the HOPG/monolayer of **1´**; **(d)** Si2p region for the HOPG/monolayer of **1´**.

3. Raman spectroscopy

Over the last few years the electrografting of aryl moieties onto C*sp*^{2} hybridized lattice surfaces such as those comprising the basal plane of graphene and HOPG surfaces,[3-4] has been successfully achieved, allowing the further characterization of these structures by Raman spectroscopy. The Raman spectrum of pristine HOPG is characterized by the G and 2D bands, whilst additional D-bands indicate the presence of defects in the C*sp*^{2} hybridized lattice surface. As displayed in Figure S2, the Raman spectrum of HOPG|1´ features characteristic bands at 1336, 1576, and 2679 cm^{-1}, which correspond to D, G, and 2D-bands, respectively.[5-6] The strong D-band (I(D)/I(G) = 0.2) indicates a significant change in the substrate structure, and demonstrates Csp3 rehybridisation of the basal plane of the HOPG associated with covalent functionalization of the surface by 1´.[3]

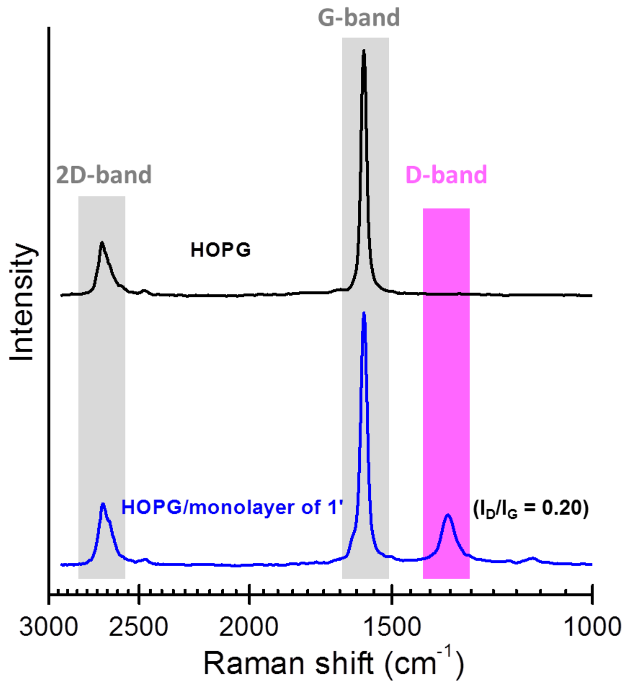

Figure S2. Raman spectra of a HOPG substrate (top) and HOPG/ 1´ (bottom).

4. Film thickness

The thickness of the electrografted monolayer of 1´ was estimated by scratching the surface with the AFM tip to reveal the underlying bare HOPG surface followed by measurement of the cross section and depth profiles across the scratched area (Figure S3). A film thickness of 2.1 ± 0.2 nm was obtained, which is in very good agreement with the

end-to-end length of the molecule (2.2 nm, Spartan 08 V1.0.0), confirming the formation of a monolayer film in which the molecules are assembled into a 2D arrangement with the molecular axis almost normal to the HOPG surface.

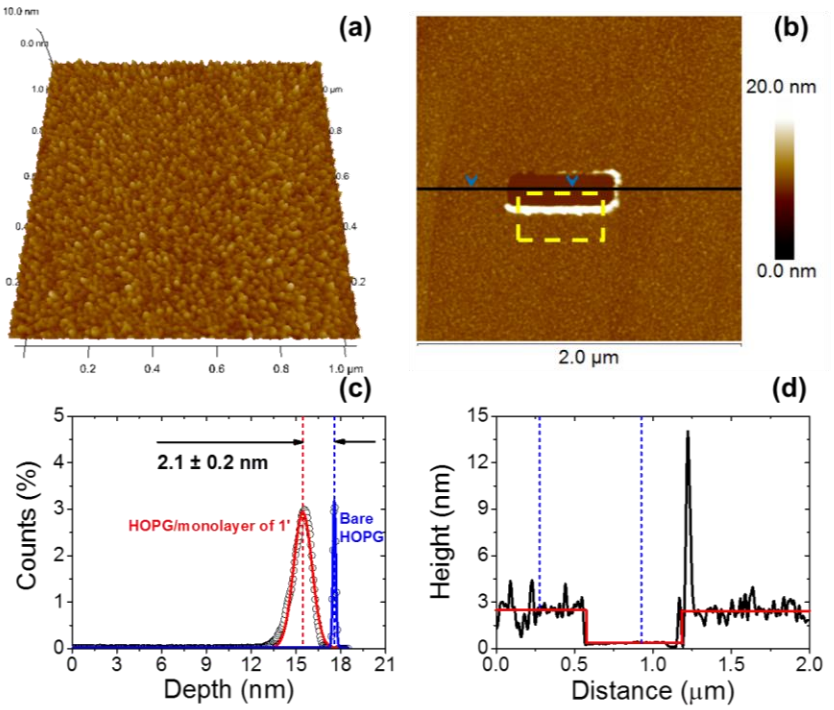

Figure S3. (a) 1.0 x 1.0 μm^2 AFM image showing the topography of a HOPG/monolayer of 1´. (b). AFM image of a 500 x 125 nm^2 scratch made in the organic film. (c) Depth profile histogram corresponding to the dashed yellow-boxed 400 x 100 nm^2 area, exhibiting the depth value distributions related to HOPG bare surface, blue line, and the organic monolayer, red line. (d) Representative cross-section profile across the scratch.

5. RMS Roughness

AFM images of the pristine HOPG substrate (Figure S4.a) feature characteristic atomically flat terraces with a number of cleavage steps and planes with an extremely low Root Mean Square (RMS) roughness value (0.07 ± 0.02 nm, Figure S4.c) as expected for a low defect C*sp*^{2} hybridized lattice surface. Similar topographic features are exhibited by the HOPG|**1**´ system (Figure S4.b) indicating the formation of a homogeneous and tightly-packed organic layer. This ability to image the characteristic features of the

underlying substrate, i.e. smooth terraces and cleavage steps is indicative of a very low density of defects, such as pinholes or 3D-protrusions of aggregates in the monolayer. The RMS roughness of HOPG|**1´** rises to 0.48 ± 0.05 nm (Figure S4.d), which is similar to the value obtained for a tightly-packed carboxylate-terminated oligo(phenylethynyl) Langmuir-Blodgett monolayer on HOPG.[7] Measurements were carried out from 30 AFM images extracted from different zones in each sample and from distinct but equivalent samples.

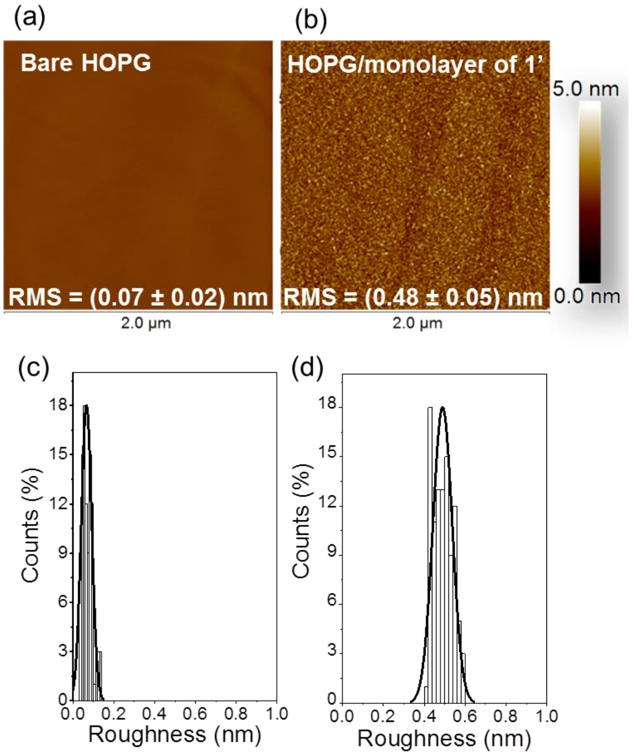

Figure S4. **(a)** 2.0 x 2.0 µm[2] AFM images showing the topography of the pristine HOPG and **(b)** HOPG/monolayer of **1´**. Histograms showing the average RMS roughness obtained for the **(c)** bare HOPG electrode and **(d)** HOPG/monolayer of **1´**.

6. AFM Characterization of C-FEBID Top Contact and Pt-FIBID Top Coat

The dimensions of two representative C-FEBID top contacts were determined by means of AFM. Figures S5a and S5b show 20 x 20 μm^2 AFM topographic images of the C-FEBID top contact and the Pt layer respectively. Figure S5c and S5d depict a representative cross section and the depth profile histogram of a C-FEBID deposit over the HOPG/monolayer of **1´** and a Pt-FIBID deposit, respectively. The referred data show that the C-FEBID deposit is (48 ± 3) nm in height and the Pt-FIBID deposit is (129 ± 5) nm in height.

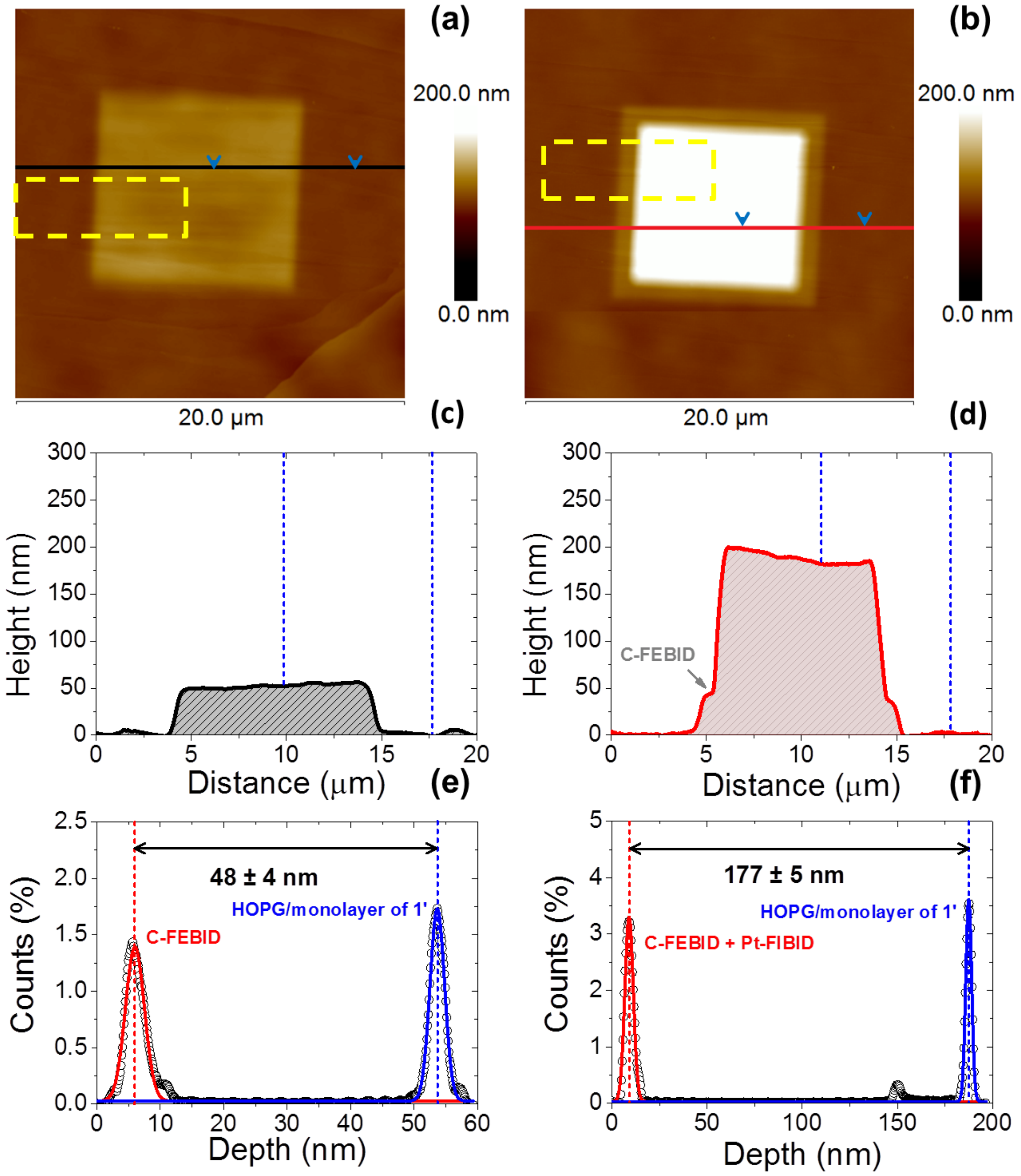

Figure S5. 20 × 20 μm^2 AFM images showing **(a)** the C-FEBID top contact and the surrounding monolayer of **1´** onto HOPG, and (b) the Pt-FIBID contact on top of the

C_FEBID and the surrounding monolayer of **1´**. **(c)** A representative cross-section profile across the squared C-FEBID stamp corresponding to black line in a. **(d)** A representative cross-section profile across the squared Pt-FIBID stamp corresponding to red line in b. **(e)** Depth profile histogram corresponding to the dashed yellow-boxed 8 × 4 μm^2 area (over one-single HOPG terrace), exhibiting the height value distributions related to both zones. From the height difference between the maxima of the depth Gaussian fits, the thickness of the C-FEBID deposit, i.e. (48 ± 4) nm, was obtained. **(f)** Depth profile histogram corresponding to the dashed yellow-boxed 8 × 4 μm^2 area (over one-single HOPG terrace), exhibiting the height value distributions related to both zones. From the height difference between the maxima of the depth Gaussian fits, the thickness of the Pt-FIBID deposit (after subtracting of the C-FEBID layer), i.e. (129 ± 5) nm, was obtained.

7. EELS Characterization of C-FEBID Top Contact

EELS experiments were performed in an FEI Titan 60-300 transmission electron microscope operated at 300 kV and fitted with a high brightness electron gun (X-FEG) and a C_S probe corrector (CETCOR), which produces an electron probe below 1 Å in STEM, and a Gatan Imaging Filter (GIF) Tridiem 866 ERS. EELS spectra acquisition was performed with an energy dispersion of 0.2 eV·pixel^{-1}, with a resolution of 0.9 eV (FWHM of the zero-loss peak), a GIF aperture of 6 mm to provide a collection angle of 55 mrad. To minimize possible beam damage, the spectra were acquired with a 40 s exposure while scanning a sample area of 10x10 nm^2. Figure S6 shows an EELS spectrum where there are no peaks attributable neither to nitrogen nor to oxygen.

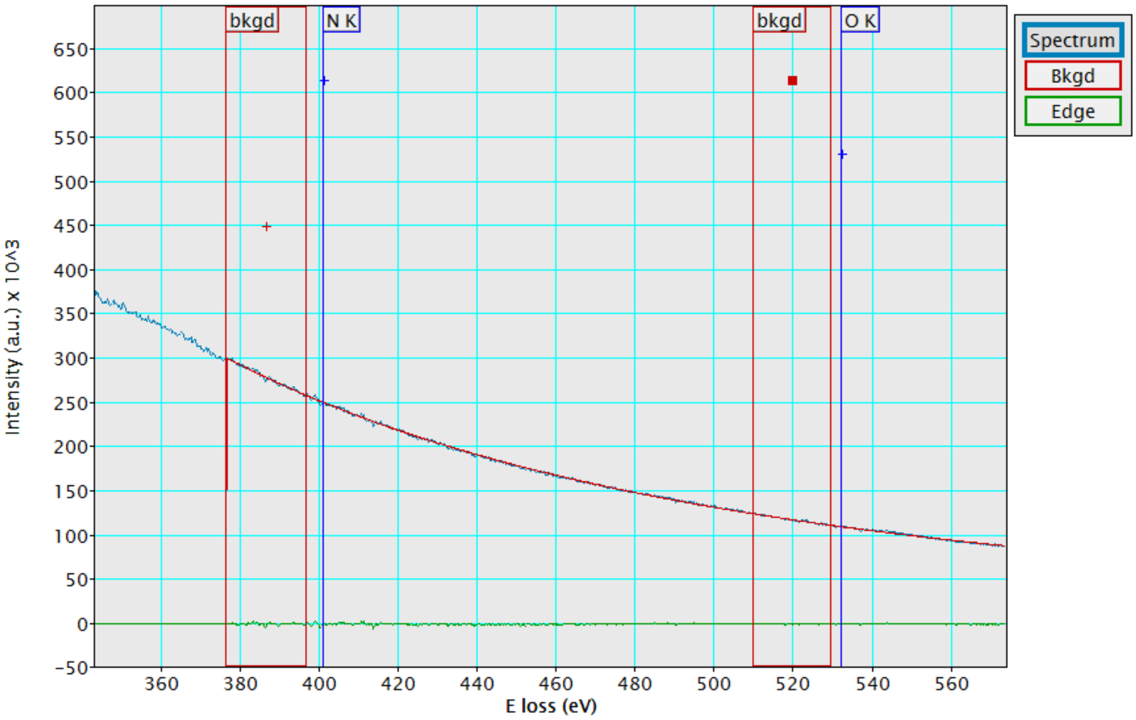

Figure S6. EELS spectra showing the N and O regions where no peak can be observed.

8. RAMAN Characterization of C-FEBID Top Contact

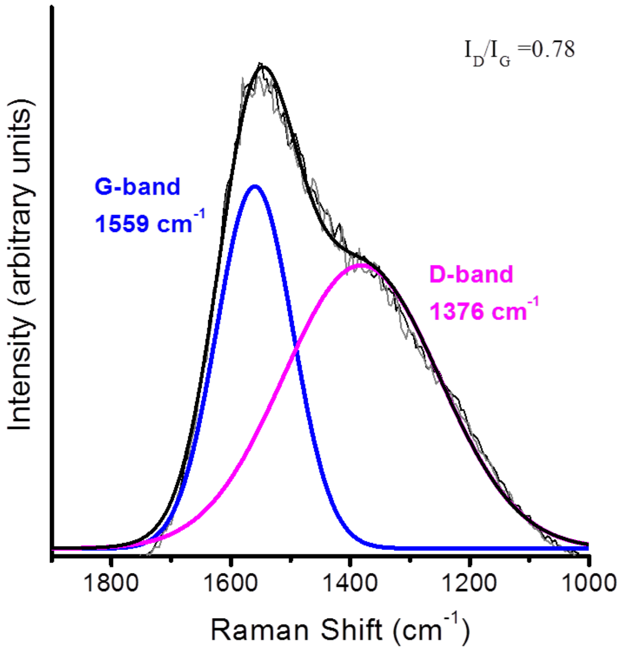

Figure S7. Raman spectra of the C-FEBID top-contact electrode.

9. Control Experiments in the *J*-*V* curves

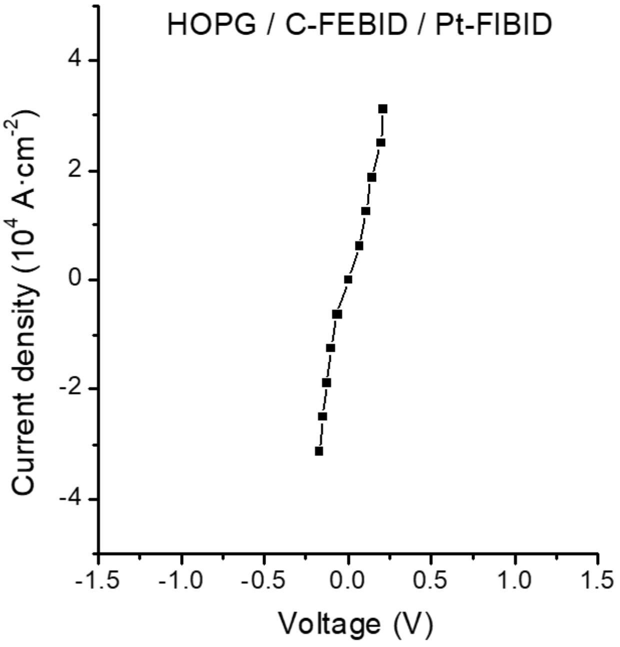

Figure S8. *J-V* curve obtained for a HOPG|C-FEBID|Pt-FIBID device (i.e., without the electrografted film between the two electrodes).

10. Simmons Fittings

J-V curves of our HOPG|**1**´|C-FEBID|Pt-FIBID devices were fitted using the Simmons equation:[8]

$$J = \frac{e}{4\pi^2 \hbar s^2}\left\{\left(\Phi - \frac{eV}{2}\right)\exp\left[-\frac{2(2m)^{1/2}}{\hbar}\alpha\left(\Phi - \frac{eV}{2}\right)^{1/2} s\right] - \left(\Phi + \frac{eV}{2}\right)\exp\left[-\frac{2(2m)^{1/2}}{\hbar}\alpha\left(\Phi + \frac{eV}{2}\right)^{1/2} s\right]\right\} \quad \text{(S1)}$$

where *s* is the width of the tunneling barrier, Φ is the effective barrier height of the tunneling junction, *V* is the potential applied to the junction, α is a parameter related to the effective mass of the electrons in the tunneling process and *e* and *m* are the charge and the mass of the electron, respectively. As a representative example, the solid red line in Figure S9 shows the best fit for a 25 μm^2 devices, with *s*, Φ and α treated as free fitting parameters. Across all devices studied, the barrier thickness values range from 2.08 to 2.15 nm, in excellent agreement with the AFM measurements (2.1 nm) performed prior to the top-contact carbon deposition.

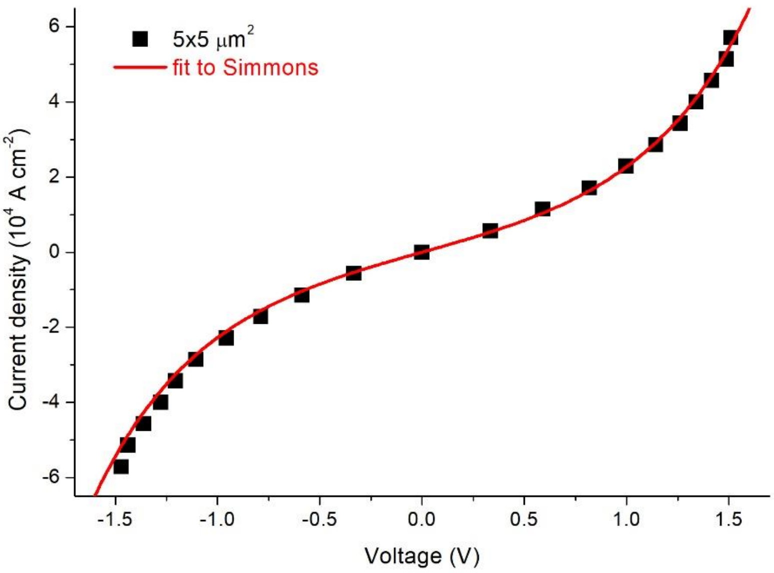

Figure S9. *J-V* characteristic of a HOPG|1'|C-FEBID|Pt-FIBID device with nominal contact area of 25 µm^2 (black squares) and its fitting to the Simmons equation with Φ = 1.59 eV, α = 0.39 and s, the thickness of the layer, 2.15 nm.

11. Theoretical Calculations

As shown in Figure 4 and S10 when the molecule is covalently bonded to both electrodes (J7 and J8) the transmission coefficient near the middle of the gap is significantly higher than when the molecule is chemically bonded to only one electrode (J5) or to neither of the electrodes (J6). Across these various models, inclusion of unsaturated and saturated defects at the contact enhances the transmission coefficient of junctions, which can be reasonably attributed to changes in the conduction paths in the molecular junction.[9]

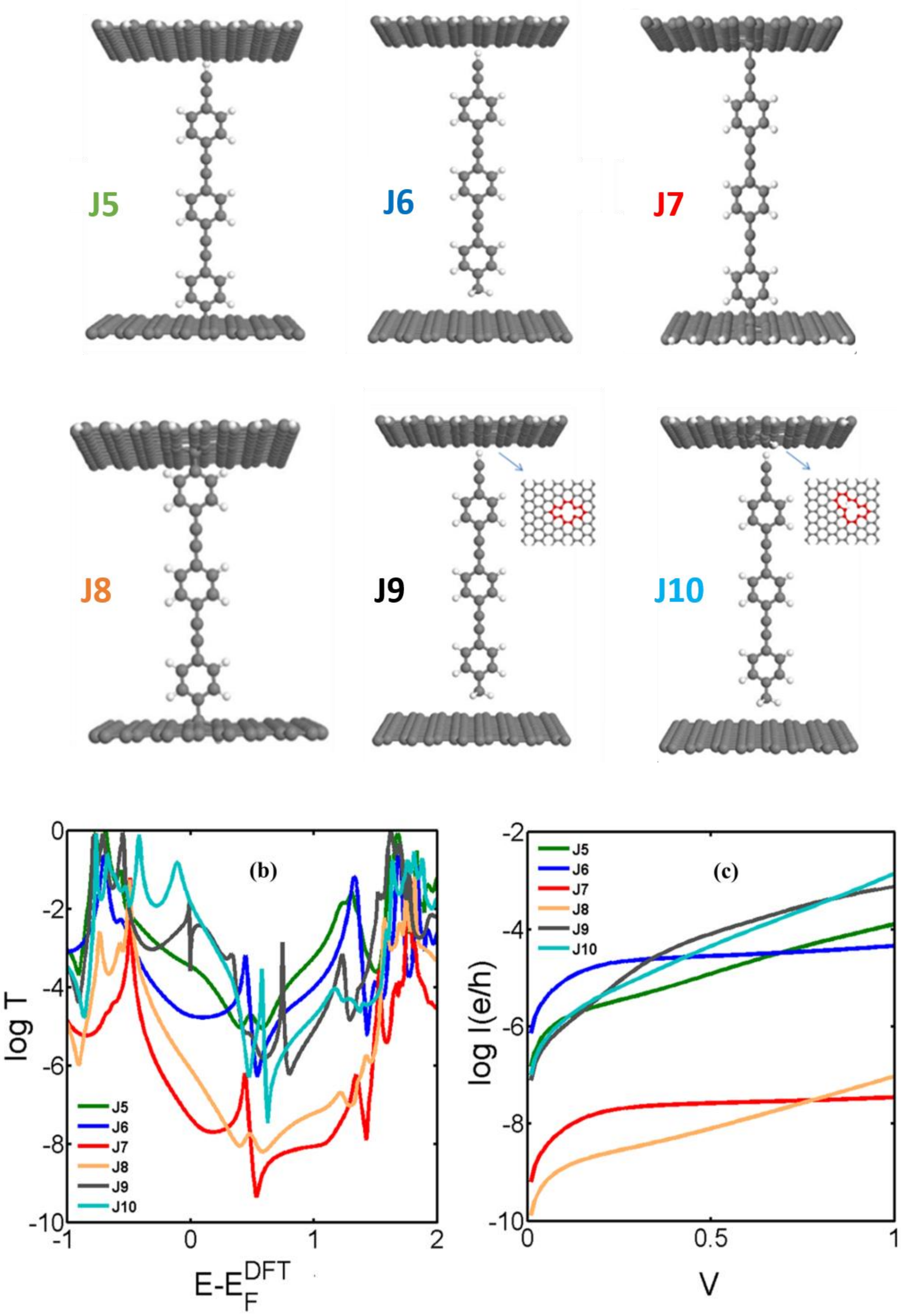

Figure S10. **(a)** Relaxed structure of the junctions: in J5 – J8 the top and bottom electrodes are modeled as idealised 2D carbon sheets (graphene-like), whilst J9 and J10 feature unsaturated and saturated defect sites near the point of molecule contact on the top electrode, respectively. In J5 the C-FEBID top-contact is physisorbed to the terminal alkyne (C≡CH); in J6, the molecule is modelled with saturated carbon fragments physisorbed to two pristine electrode surfaces; in J7, the molecule is covalently bound to each electrode; in J8, the molecule is covalently bound to each

electrode with the ethynyl moiety incorporated into the C-FEBID top-contact; J9 and J10 are derived from J6, with unsaturated and saturated defects introduced into the top contact electrode, respectively. Plots of the: **(b)** transmission coefficients; and **(c)** calculated current-voltage relations of J5 – J10.

In the simulations shown in Figure 4 and Figure S10, the edges of the electrodes were fully saturated, which corresponds to the most likely, chemically reasonable form of the carbon deposits. We also studied the effect of unsaturated carbons at the edges of the electrodes. As shown by Figure S10, the results are very similar to the calculation with fully saturated, hydrogen-terminated edges (Figure 4b and S10b), except a new feature close to the Fermi energy appears, which vanishes when edges of the electrodes are saturated.

In order to understand the effect of edge termination and boundary condition on transport properties, we constructed all junctions described in the paper without hydrogen termination and with the periodic boundary condition. As it is illustrated in Figure S11, the results are very similar to previous calculation with H termination, except for a new feature close to the Fermi energy, which vanishes when the edges of the electrodes are hydrogen terminated.

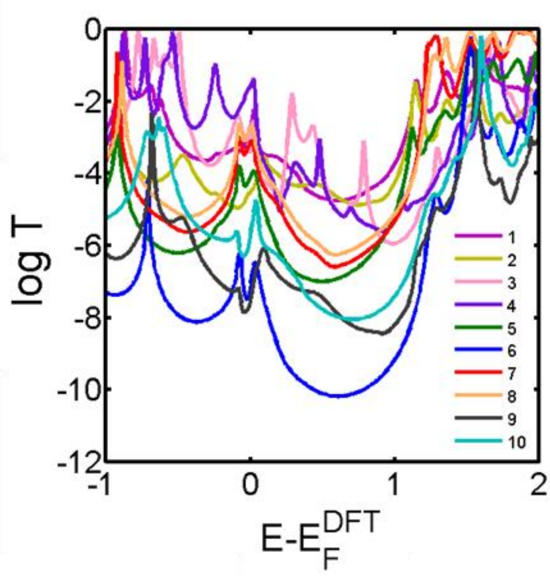

Figure S11. Transmission coefficient T corresponds to the junctions J1 to J10 without H-terminated electrode.

12. References

1. [illegible]
2. [illegible]
3. [illegible]
4. [illegible]
5. [illegible]
6. [illegible]
7. [illegible]
8. [illegible]
9. [illegible]
